# Ghrelin in the human myometrium

**DOI:** 10.1186/1477-7827-8-55

**Published:** 2010-05-28

**Authors:** Margaret O'Brien, Padraig Earley, John J Morrison, Terry J Smith

**Affiliations:** 1 National Centre for Biomedical and Engineering Science, Orbsen Building, National University of Ireland Galway, University Road, Galway, Ireland; 2 Department of Obstetrics and Gynaecology, National University of Ireland Galway, Clinical Science Institute, University College Hospital Galway, Newcastle Road, Galway, Ireland

## Abstract

**Background:**

Ghrelin is a 28-amino acid octanolyated peptide, synthesised primarily in the stomach. It stimulates growth hormone release, food intake and exhibits many other diverse effects. Our group have previously determined that ghrelin inhibited human contractility in *vitro*. The aim of this study therefore, was to investigate the expression of ghrelin, its receptor, the growth hormone secretagogue receptor type 1 (GHS-R1), ghrelin O-acyltransferase (GOAT) which catalyses ghrelin octanoylation, prohormone convertase 1/3 (PC1/3) responsible for pro-ghrelin processing, in human myometrium, during pregnancy prior to labour, during labour and in the non-pregnant state. Modulation of ghrelin and ghrelin receptor expression in cultured myometrial cells was also investigated.

**Methods:**

mRNA and protein were isolated from human myometrium and the myometrial smooth muscle cell line hTERT-HM; and real-time fluorescence RT-PCR, western blotting and fluorescence microscopy performed. The effects of β-Estradiol and bacterial lipopolysaccharide (LPS) on hTERT-HM gene expression were evaluated by western blotting.

**Results:**

We have reported for the first time the expression and processing of ghrelin, GHS-R1, GOAT and PC1/3 expression in human myometrium, and also the down-regulation of ghrelin mRNA and protein expression during labour. Furthermore, GHS-R1 protein expression significantly decreased at labour. Myometrial GOAT expression significantly increased during term non-labouring pregnancy in comparison to both non-pregnant and labouring myometrium. Mature PC1/3 protein expression was significantly decreased at term pregnancy and labour in comparison to non-pregnant myometrium. Ghrelin, GHS-R1, GOAT and PC1/3 mRNA and protein expression was also detected in the hTERT-HM cells. Ghrelin protein expression decreased upon LPS treatment in these cells while β-Estradiol treatment increased GHS-R1 expression.

**Conclusions:**

Ghrelin processing occurred in the human myometrium at term pregnancy and in the non-pregnant state. GOAT expression which increased during term non-labouring pregnancy demonstrating a similar expression pattern to prepro-ghrelin and GHS-R1, decreased at labour, signifying possible myometrial ghrelin acylation. Moreover, the presence of PC1/3 may contribute to pro-ghrelin processing. These results along with the previous *in vitro *data suggest that myometrially-produced and processed ghrelin plays a significant autocrine or paracrine role in the maintenance of relaxation in this tissue during pregnancy. Furthermore, the significant uterine modulators LPS and β-Estradiol are involved in the regulation of ghrelin and ghrelin receptor expression respectively, in the human myometrium.

## Background

Ghrelin is an acylated 28-amino acid hormone [1] synthesised primarily in the stomach, it is also found in a wide range of other tissues including the hypothalamus, placenta, ovary, testis and endometrium [2,3]. Ghrelin has an *N*-octanoyl group covalently linked to the hydroxyl group of its serine-3 residue, this modification being necessary for ghrelin to bind to its receptor, the growth hormone secretagogue receptor (GHS-R1) [4]. No other naturally occurring peptide has been identified to have this post-translational acyl group modification [5]. It is through the mediation of the seven transmembrane G-protein coupled receptor GHS-R1, coupled to the G_q _subunit that ghrelin elicits growth hormone release, regulates appetite and energy metabolism and performs many functions in other tissues and organs, such as the reproductive system. In obese non-pregnant adults ghrelin levels are reduced but are increased in subjects of low body mass index [6]. GHS-R1 is primarily expressed in the pituitary and the hypothalamus but is also widely expressed in many tissues including the stomach, testis, ovaries and endometrium [3,7]. The enzyme that catalyses the octanoylation of ghrelin in the endoplasmic reticulum, is ghrelin *O*-acyltransferase (GOAT) [8,9]. GOAT or mBOAT4 is a member of the family of membrane-bound *O*-acyltransferases. Des-acyl or unacylated ghrelin is the dominant form of ghrelin in the plasma, it cannot bind the GHS-R1 receptor but it has been suggested it plays alternative roles in a GHS-R1 independent pathway [5,10].

The prepro-ghrelin protein is 117-amino acids in length: the signal peptide is cleaved to form the 94-amino acid pro-ghrelin peptide. A pro-hormone convertase 1/3 (PC1/3) is the only enzyme known to be responsible for the conversion of pro-ghrelin to ghrelin *in vivo *[11], it cleaves the 94-amino acid human ghrelin precursor into the 28-amino acid mature ghrelin, through proteolytic dibasic cleavage at the LQPR/ALAG site [12]. PC1/3 is also capable of processing other hormones such as oxytocin [13], in dense core granules of the regulated secretory pathway [14].

In human pregnancy, total serum ghrelin levels peak around mid-gestation and fall to their lowest levels in the third trimester, and increase again postpartum [15,16]. Acylated ghrelin levels were significantly increased in the 2^nd^, and decreased in the 3^rd ^trimester compared to non-pregnant samples and samples taken during 1^st ^trimester of pregnancy [17]. Moreover, acylated ghrelin markedly decreased during pregnancy compared to the postpartum period [18]. A previous report from our group highlighted that ghrelin had significant inhibitory effects on *in vitro *myometrial contractility [19].

Our aim therefore was to determine if ghrelin was expressed in the myometrium, and if so, investigate its regulation in this tissue during pregnancy. Firstly, we investigated ghrelin expression in the human myometrium during pregnancy, at term in labour (PL), term not in labour (PNL) and in the non-pregnant (NP) state. Secondly, the myometrial expression of GHS-R1, GOAT and PC1/3 was examined. Thirdly, we studied the effects of two compounds that regulate myometrial function, β-Estradiol and LPS, on ghrelin and GHS-R1 expression in a human myometrial smooth muscle cell line, hTERT-HM [20].

## Methods

### Tissue collection

Biopsies of human myometrial tissue during pregnancy were obtained at elective (pregnant not in labour, PNL, i.e. prior to labour) and intrapartum (pregnant in labour, PL) caesarean section operations. The biopsies were excised from the upper lip of the lower uterine segment incision in the midline, i.e. the upper portion of the lower uterine segment. Women who had received prostaglandins or oxytocin for either induction or augmentation of labour were excluded from the study. Biopsies of human non-pregnant myometrial tissue (non-pregnant, NP) were obtained from pre-menopausal hysterectomy specimens, and women receiving therapy with progestagens, luteinising hormone-releasing hormone analogues, or steroid based medications were excluded from the study. Women with gynaecologic malignancy were also excluded from the study. Myometrial samples were carefully dissected to minimise decidual inclusion. Ethical Committee approval for the tissue collection procedure was obtained from the Research Ethics Committee University College Hospital, Galway and recruitment was by written informed consent. Immediately upon collection, tissue was snap-frozen in liquid nitrogen, and stored at -80°C.

### Tissue samples for real-time fluorescence RT-PCR

PNL (*n *= 3), PL (*n *= 3) and NP (*n *= 3) samples were obtained for real time RT-PCR. The reasons for elective caesarean section included previous caesarean section (*n *= 2) and placenta praevia (*n *= 1). The reasons for emergency caesarean section were face presentation (*n *= 1), appendectomy (*n *= 1) and previous classical caesarean section (*n *= 1). The patient demographic data for the PNL and PL samples is summarised in Table 1. The reasons for hysterectomy were prolapse (*n *= 1), fibroids (*n *= 1) and menorrhagia (*n *= 1), the average age of the hysterectomy samples was 42 (range 41-43).

**Table 1:** Real time RT-PCR patient demographic data

Pregnancy State	NL (*n *= 3)	L (*n *= 3)
*Mean Age at Delivery (years) +/- SEM*	32.7 ± 1.8	33.7 ± 3.7
*Range*	30-36	29-41
*Parity*	0: 1 woman 1: 2 women ≥ 2: 0 women	0: 1 woman 1: 1 woman ≥ 2: 1 woman
*Mean Gestation Length (weeks) +/- SEM*	38.7 ± 0.33	38.1 ± 1.1
*Range*	38-39	37-40.3
*Mean Baby Birth-weight (kg) +/- SEM*	3.45 ± 0.06	3.7 ± 0.17
*Range*	3.0-3.59	3.32-4.48

### Tissue samples for protein expression

PNL (*n *= 5), PL (*n *= 6) and NP (*n *= 8) samples were obtained for western blotting. The reasons for hysterectomy were menorrhagia (*n *= 5), prolapse (*n *= 2) and fibroids (*n *= 1), the average age was 44.5 (range 33-54). The reasons for elective caesarean section included previous caesarean section(s) (*n *= 4) and poor obstetric history (*n *= 1). The reasons for emergency caesarean section were failure to progress/advance (*n *= 3), face presentation (*n *= 1), non-reassuring foetal testing (*n *= 2). The patient demographic data for the PNL and PL samples is presented in Table 2.

**Table 2:** Western blot patient demographic data

Pregnancy State	NL (*n *= 5)	L (*n *= 6)
*Mean Age at Delivery (years) +/- SEM*	28.7 ± 1.9	35.2 ± 1.9
*Range*	25-33	28-39
*Parity*	0: 0 women 1: 2 women ≥ 2: 3 women	0: 2 women 1: 4 women ≥ 2: 0 women
*Mean Gestation Length (weeks) +/- SEM*	38.2 ± 0.14	40.4 ± 0.3
*Range*	38-38.7	39.6-41
*Mean Baby Birth-weight (kg) +/- SEM*	3.3 ± 0.1	3.85 ± 0.2
*Range*	3.0-3.59	3.32-4.48

### Chemicals

Human ghrelin (octanoylated), leptin, LPS, oxytocin, water soluble β-Estradiol and progesterone were purchased from Sigma (Dublin, Ireland).

### Cell culture

Myometrial human telomerase reverse transcriptase (hTERT-HM) cells kindly provided by Dr. Jennifer C. Condon [20] were cultured in DMEM-F-12/10% FBS (Invitrogen, Carlsbad, CA, USA). Prior to treatment for western blotting, cells were pre-conditioned overnight in 2-10% charcoal stripped FBS (Biosera, East Sussex, UK) and phenol red free DMEM-F12 (Invitrogen, USA) and β-estradiol and LPS experiments performed in serum-free phenol red-free medium for 16 hours.

### RNA extraction and reverse transcription

Total RNA was isolated from tissue using TRIzol reagent (Life Technologies Ltd., UK) [21] and cells using the RNeasy mini RNA isolation kit (Qiagen, Crawley, West Sussex, UK). RNA (1 μg- DNase I treated) was reverse transcribed for use as a template for Polymerase Chain Reaction (PCR). Samples in which no reverse transcriptase was added were included to confirm that no genomic DNA contamination was present.

### Real time fluorescence PCR

Real time fluorescence PCR was performed using the Applied Biosystems StepOne Plus™ Real Time PCR System Relative Standard Curve method (ABI, Foster City, CA, USA) [22]. The sequence of oligonucleotide primers (MWG, Ebersberg, Germany) for real time PCR were:

*GHRL *(Ghrelin)

5'-TGAGCCCTGAACACCAGAGAG-3'

5'-AAAGCCAGATGAGCGCTTCTA-3' [23]

GHSR

5'-AGCGCTACTTCGCCATC-3'

5'-CCGATGAGACTGTAGAG-3' [24]

*GHSR*a and b

R1F 5'-TCGTGGGTGCCTCGCT-3'

R1aR 5'- CACCACTACAGCCAGCATTTC-3'

R1bR 5'- GCTGAGACCCACCCAGCA-3' [25]

MBOAT4

5'- CATCTGGACCCTGGAAA-3'

5'-CGAAACCAAACACCTGTC-3' XM_001717299.1

*PCSK1 *(PC1/3)

5'-TGTTCACACATGGGGAG-3'

5'-ACGAGGCTGCTTCATATG-3' [26]

ACTB

5'-GGGCATGGGTCAGAAGGATT-3'

5'-AGTTGGTGACGATGCCGTG-3' (Accession M10277)

Each reaction was performed in triplicate. 2-tailed unpaired student t-tests were performed using Graphpad Prism 5 software (GraphPad Software, Inc., USA). P values < 0.05 were considered to be statistically significant.

### Protein isolation-tissue

Human myometrial tissue was homogenised and prepared as described previously [27].

### Protein isolation-cells

Cell pellets after trypsinisation were washed in 1×PBS, centrifuged and re-suspended in ice-cold lysis buffer [28].

### Western blot analysis

Proteins were resolved by SDS (w/v) polyacrylamide gel electrophoresis gels (BioRad, Hercules, CA, USA) as previously described [29]. Membranes were blocked and incubated overnight at 4°C with 1/50-1/100 dilution of anti-human ghrelin rabbit polyclonal primary antibody (sc-50297), 1/100 dilution of ghrelin goat polyclonal (sc 10368), 1/100-1/200 GHS-R1 rabbit polyclonal (sc20748) (Santa Cruz Biotechnology Inc., Rockford, IL, USA), 1/500 GOAT rabbit (H-032-12) (Phoenix Pharmaceuticals Inc. Beach Road, Burlinghame, CA, USA), 1/500 PC1/3 mouse monoclonal (ab55543) (Abcam plc, Milton Road, Cambridge, UK) or 10,000 dilution of β-actin (ACTB) mouse monoclonal AC-15 (Sigma-Aldrich, Ireland) antibodies. Blots were washed and incubated in either 1:4,000 dilution of rabbit anti-goat (DakoCytomation Ltd, UK), 1:1000 goat anti-rabbit or 1:1000 dilution goat anti-mouse horseradish peroxidase-conjugated secondary antibodies (Pierce Technology, USA), the bound secondary antibody was detected as previously described [29]. Statistical analysis of the densitometric data for each protein band compared to β-Actin (one way ANOVA with Tukey's post hoc analysis or 2-tailed unpaired student t-tests) and graph construction were performed using GraphPad Prism version 4 (GraphPad Software Inc., USA).

### Immunofluorescence microscopy

Cells were prepared as described previously and incubated with primary antibodies to ghrelin, GHS-R1, GOAT or PC1/3 [29].

## Results

### Real time fluorescence RT-PCR-Tissue

#### Ghrelin

**Figure 1:**
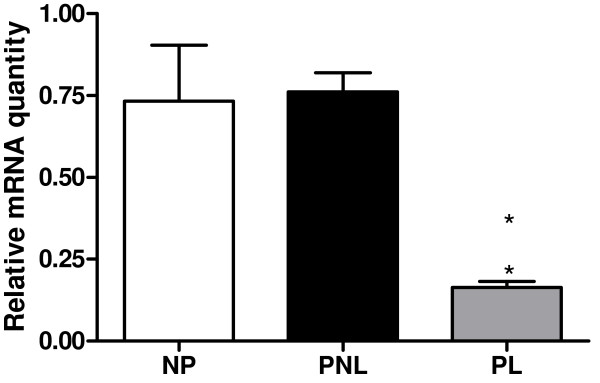
**Graphical representation of real-time fluorescence RT-PCR results of β-Actin-normalised relative ghrelin mRNA quantity plotted against myometrial pregnancy state**. NP (*n *= 3), PNL (*n *= 3), PL (*n *= 3) ± SEM (indicated by the error bars). * indicates a significance value of P < 0.05 between NP v PL and PNL v PL.

There was a significant decrease in ghrelin expression in PL, in comparison to NP (4.47 fold decrease; *P *< 0.05), and in comparison to PNL myometrium (4.65-fold decrease: *P *< 0.05) (Figure 1). The corresponding densitometric data is presented in Table 3. Total GHS-R1, GHS-R1a, GHS-R1b, GOAT and PC1/3 mRNA expression was detected; however there was no significant change in expression of these genes in the myometrium in any of the 3 conditions, NP, PNL or PL (data not shown).

**Table 3:** Summary of tissue mRNA and protein densitometry data

	Non-Pregnant (NP) (Densitometric value ± SEM)	Pregnant not in labour (PNL) (Densitometric value ± SEM)	Pregnant in labour (PL) (Densitometric value ± SEM)
**Ghrelin mRNA (Figure 1)**	0.733 ± 0.171	0.761 ± 0.058	0.164 ± 0.018
**Ghrelin protein (Figure 2)**			
Prepro-ghrelin (i)	0.655 ± 0.094	0.557 ± 0.022	0.375 ± 0.049
Pro-ghrelin (ii)	0.349 ± 0.070	0.301 ± 0.039	0.352 ± 0.075
Ghrelin (iii)	0.574 ± 0.061	0.457 ± 0.054	0.300 ± 0.091
**Ghrelin protein (Figure 3)**			
Prepro-ghrelin (i)	1.578 ± 0.441	1.280 ± 0.092	0.521 ± 0.050
Pro-ghrelin (ii)	0.304 ± 0.068	0.677 ± 0.151	1.162 ± 0.421
**GHS-R1 protein (Figure 4)**	0.524 ± 0.053	0.589 ± 0.040	0.364 ± 0.054
**GOAT protein (Figure 5)**	0.428 ± 0.040	0.740 ± 0.073	0.311 ± 0.051
**PC1/3 protein (Figure 6)**	0.174 ± 0.049	0.061 ± 0.005	0.038 ± 0.004

### Westerns-tissue

#### Ghrelin

**Figure 2:**
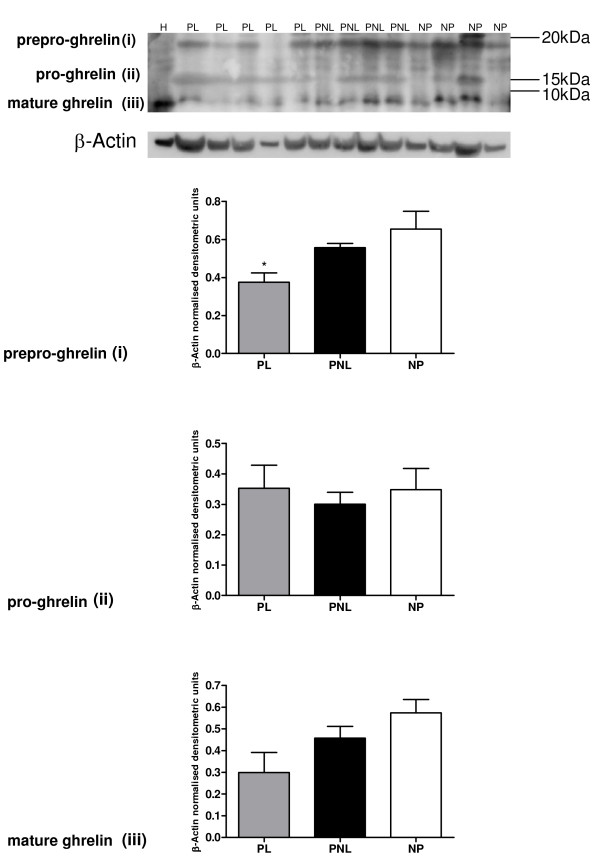
**A representative western blot of ghrelin (sc anti-rabbit antibody) protein expression in human myometrium**. NP (*n *= 4), PNL (*n *= 4), PL (*n *= 5) and HeLa cells (H). (i) Prepro-ghrelin and (ii) pro-ghrelin and (iii) mature ghrelin bands are indicated. The corresponding β-Actin (ACTB) western blot is presented underneath each blot. Molecular weights are indicated in kDa. Quantitative densitometric analysis of each ghrelin isoform is presented below the relevant blot(s) (i-iii), with β-Actin normalised densitometric units for each protein plotted against pregnancy state ± SEM (indicated with error bars). * indicates a significance value of P < 0.05. Significance values indicated are compared to NP tissue expression.

**Figure 3:**
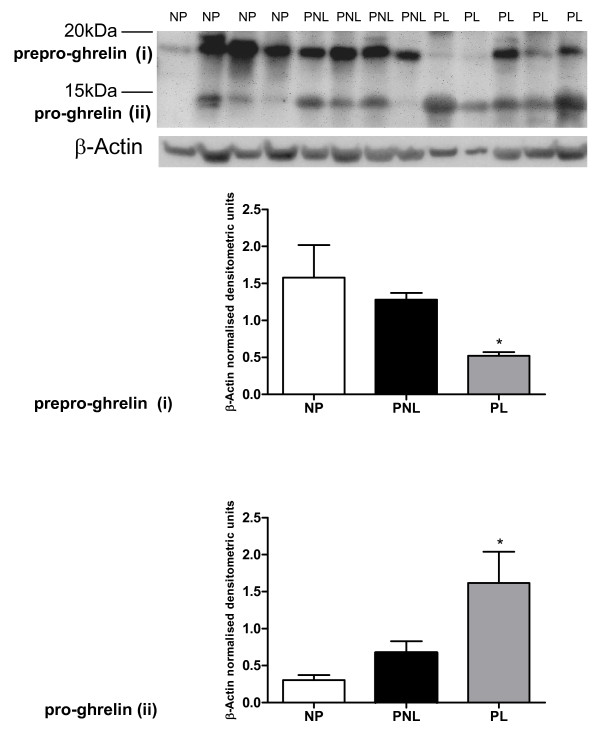
**A representative western blot of ghrelin (sc anti-goat antibody) protein expression in human myometrium**. NP (*n *= 4), PNL (*n *= 4) and PL (*n *= 5) (i) prepro-ghrelin and (ii) pro-ghrelin and (iii) mature ghrelin bands are indicated. The corresponding β-Actin (ACTB) western blot is presented underneath each blot. Molecular weights are indicated in kDa. Quantitative densitometric analysis of each ghrelin isoform is presented below the relevant blot(s) (i-ii), with β-Actin-normalised densitometric units for each protein plotted against pregnancy state ± SEM (indicated with error bars). * indicates a significance value of P < 0.05. Significance values indicated are compared to NP tissue expression.

Ghrelin expression was observed in the human myometrial tissue (Figures 2 and 3) and in HeLa cells, which were used as a positive control (Figure 2). Protein bands of the expected sizes for prepro-ghrelin (approximately 18 kDa), pro-ghrelin (13 kDa) and mature ghrelin (5-6 kDa) protein were visualised by western blotting in the samples. Two ghrelin antibodies were used for these tissue westerns, rabbit (sc50297) (Figure 2) and goat (sc10368) (Santa Cruz, USA) (Figure 3). All 3 ghrelin isoforms, the prepro-, the pro- and the mature ghrelin were visible on the western blots however, the goat antibody had difficulty hybridising to the mature human ghrelin isoform (Figure 3). There was a significant decrease of at least 75% (*P *< 0.05) in expression of the prepro- form in the PL samples in comparison to the non-pregnant state, using both antibodies (Figures 2(i) and 3(i)). An increase (quantified by densitometric analysis to be significant, using the goat antibody) was evident in pro-ghrelin protein levels at labour, compared to non-pregnant tissue (*P *< 0.05) (Figure 3(ii)). The corresponding densitometric data is presented in Table 3.

#### GHS-R1

**Figure 4:**
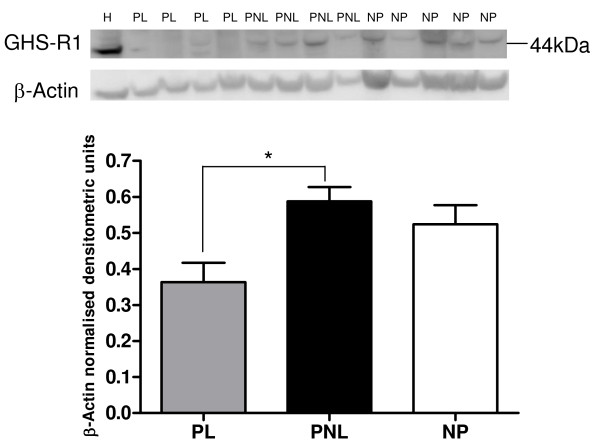
**A representative Western blot of GHS-R1 protein expression in human myometrial tissue samples**. PL (*n *= 4), PNL (*n *= 4) and NP (*n *= 5), and HeLa cells (H). The corresponding ACTB protein expression is presented underneath. Molecular weights are indicated in kDa. Quantitative densitometric analysis of the western blot is presented, with β-Actin-normalised densitometric units for GHS-R1 plotted against pregnancy state ± SEM (indicated with error bars). * indicates a significance value of P < 0.05.

A weak band of the expected size of approximately 44 kDa was observed in the human myometrial tissue and also in the positive control HeLa cell lysate using an antibody to the ghrelin receptor (Figure 4). There was a decrease in the expression of GHS-R1 in the PL versus those of the PNL and the NP protein samples, with a significant decrease of 62% (*P *= 0.015) in the PNL compared to the PL samples (Figure 4 and Table 3).

#### GOAT

**Figure 5:**
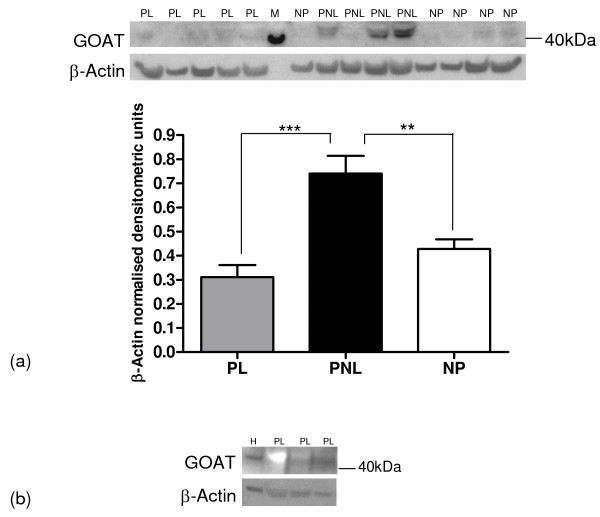
**Representative Western blots of GOAT protein expression in human myometrium**. (a) PL (*n *= 5), PNL (*n *= 4), NP (*n *= 5), (b) HeLa cells (H) (*n *= 1) and myometrial PL (*n *= 3). The corresponding ACTB western blots are presented underneath each western blot. Molecular weights (M) are indicated in kDa. Quantitative densitometric analysis of each protein band indicated a(i-iii) is presented, with β-Actin-normalised densitometric units for GOAT plotted against pregnancy state ± SEM (indicated with error bars). ** indicates a significance value of P < 0.01, *** indicates a significance value of P < 0.001.

GOAT protein expression was observed in the tissue samples and in the HeLa cells (Figure 5a and 5b). A weak band of 40-45 kDa was observed, which is slightly lower than the expected size of 50 kDa. There was a significant increase in GOAT protein expression at term pregnancy compared to the non-pregnant (73%, P < 0.01) and the labouring myometrial (137%, P < 0.001) samples (Figure 5a and Table 3).

#### PC1/3

**Figure 6:**
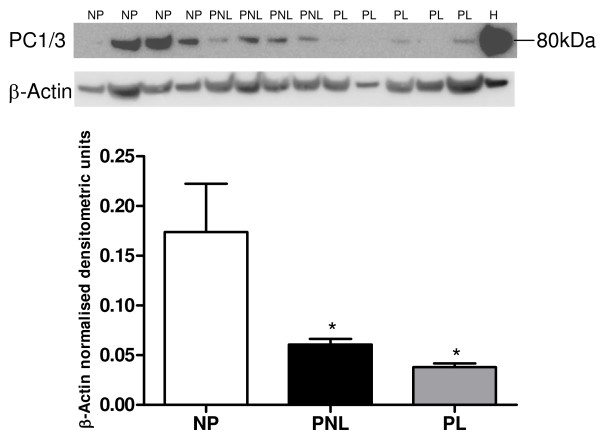
**A representative Western blot of PC1/3 protein expression in human myometrium**. NP (*n *= 4), PNL (*n *= 4), PL (*n *= 5) and HeLa cells (H). The corresponding ACTB western blots are presented underneath. Molecular weights (M) are indicated in kDa. Quantitative densitometric analysis of the western blot is presented, with β-Actin-normalised densitometric units for PC1/3 plotted against pregnancy state ± SEM (indicated with error bars). * indicates a significance value of P < 0.05, compared to the NP tissue.

PC1/3 protein expression was observed in NP, PNL and PL tissue and in the HeLa cell lysate (Figure 6). The only band that was observed in all cases using the Abcam monoclonal antibody was the mature PC1/3 (with the signal sequence cleaved), the expected size was 84 kDa (Abcam, UK). Mature PC1/3 expression was found to be high in the NP samples and significantly reduced, with a 2.85 (*P *< 0.05) and a 4.58 (*P *< 0.05) fold-change compared to PNL and PL respectively. Furthermore, there was a slight reduction in PC1/3 expression at labour compared to term pregnancy (Figure 6 and Table 3).

### Westerns-cells

#### Ghrelin

**Figure 7:**
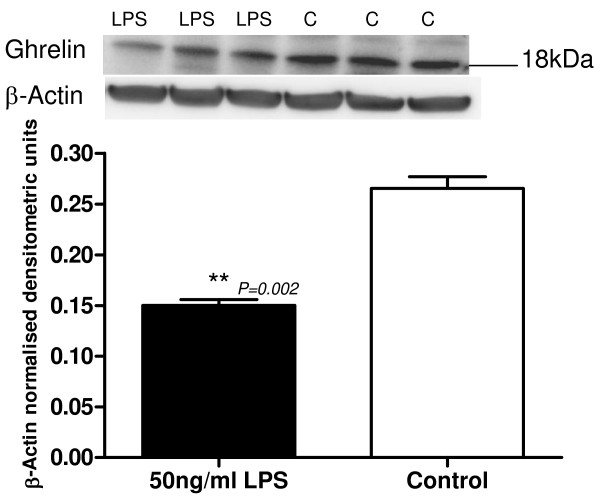
**Representative Western blot of prepro-ghrelin protein expression in LPS (50 ng/ml) treated hTERT-HM cells compared to control (C) untreated cells**. The corresponding ACTB western blot is presented underneath the western blot. Molecular weights are indicated in kDa. Quantitative densitometric analysis of this western blot is presented, with β-Actin-normalised densitometric units for prepro-ghrelin plotted against treatment ± SEM (indicated with error bars). * indicates a significance value of P < 0.05, ** indicates a significance value of P < 0.01.

Ghrelin expression was monitored in the hTERT-HM cells after 16 hour exposure to 50 ng/ml LPS, in serum-free and phenol red-free medium. The anti-goat ghrelin antibody was used for the cell westerns, and all ghrelin western densitometric analyses were performed on the prepro-ghrelin band. There was a significant decrease in the expression of prepro-ghrelin protein of 77% (*P *= 0.002) with 50 ng/ml LPS (Figure 7).

#### GHS-R1

**Figure 8:**
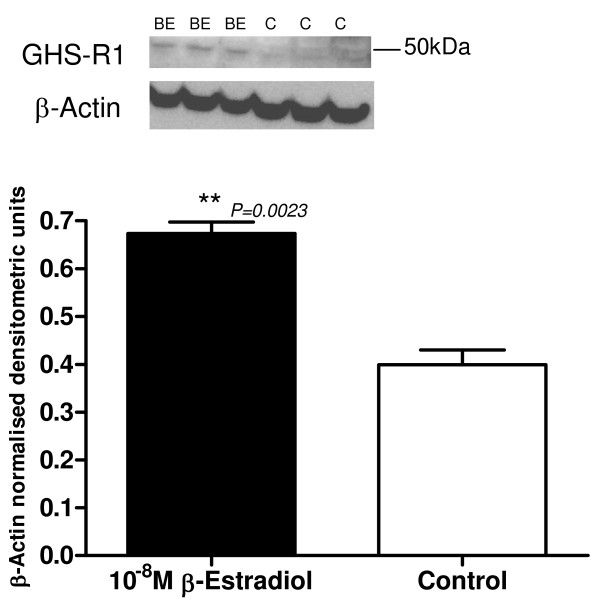
**Representative Western blot of GHS-R1 protein expression of β-Estradiol 10^-8^M (BE) treated hTERT-HM cells compared to control (C) untreated cells**. The corresponding ACTB western blot is presented underneath the western blot. Molecular weights are indicated in kDa. Quantitative densitometric analysis of this western blot is presented, with β-Actin-normalised densitometric units for prepro-ghrelin plotted against treatment ± SEM (indicated with error bars). ** indicates a significance value of P < 0.01.

GHS-R1 protein expression increased after 16 hour exposure to 10^-8^M β-Estradiol in serum-free phenol red-free medium, in comparison to control untreated hTERT-HM cells (Figure 8). There was a significant 2-fold increase in GHS-R1 protein expression after 10^-8^M β-estradiol treatment (*P *= 0.0023).

#### GOAT and PC1/3

**Figure 9:**
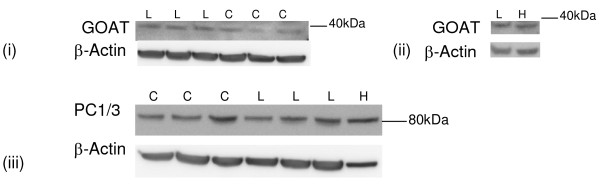
**Representative Western blots of (i and ii) GOAT protein expression (iii) PC1/3 expression in hTERT-HM cells and in HeLa cells (H)**. The corresponding ACTB western blot is presented underneath. Molecular weights are indicated in kDa.

GOAT and PC1/3 expression was observed in the hTERT-HM cell line and also in the HeLa cells. A representative hTERT-HM cell western blot for each protein is presented in Figure 9 with (i and ii) cells analysed for GOAT expression and (iii) cell proteins hybridised to the PC1/3 antibody.

### Fluorescence microscopy

#### Ghrelin, GHS-R1, GOAT, PC1/3

**Figure 10:**
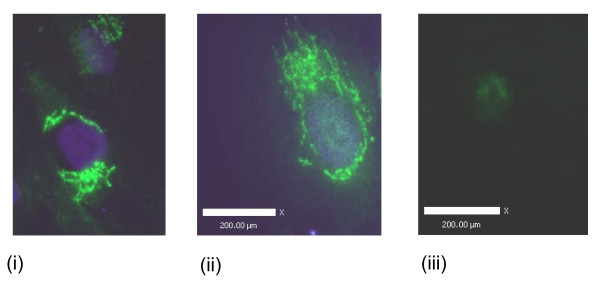
**Representative immunofluorescence images of ghrelin localisation in human myometrial smooth muscle cells (hTERT-HM)**. The FITC negative secondary antibody control is presented (iii) and scale bars are included.

**Figure 11:**
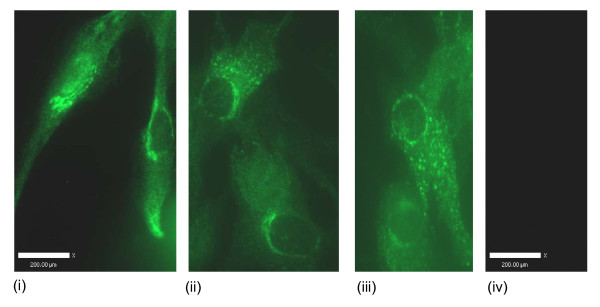
**Representative immunofluorescence images of GHS-R1 localisation in human myometrial smooth muscle cells (hTERT-HM)**. The FITC negative secondary antibody control is presented (iv) and scale bars are included.

**Figure 12:**
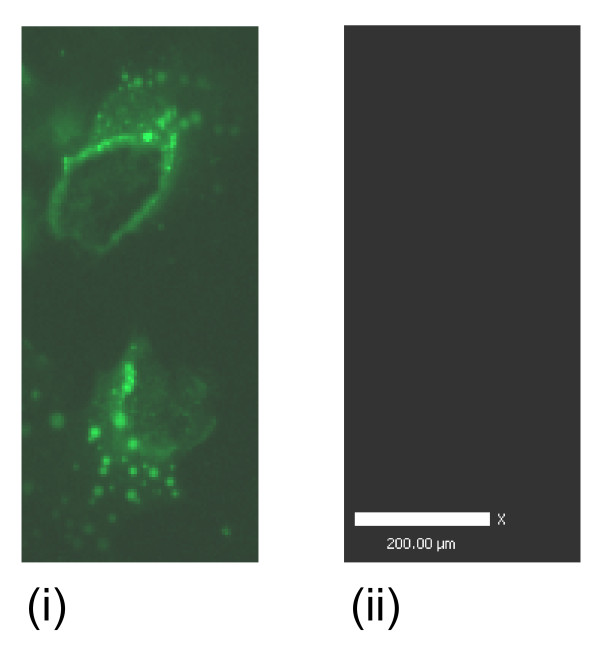
**Representative immunofluorescence images of GOAT localisation in human myometrial smooth muscle cells (hTERT-HM)**. The FITC negative secondary antibody control is presented (ii) and a scale bar is included.

**Figure 13:**
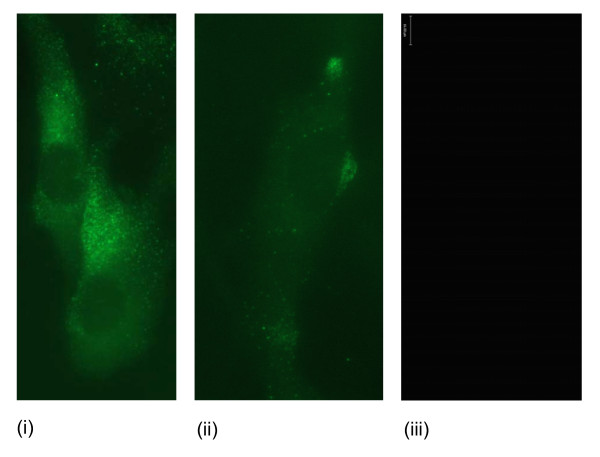
**Representative immunofluorescence images of PC1/3 localisation in human myometrial smooth muscle cells (hTERT-HM)**. The FITC negative secondary antibody control is presented (iii).

Ghrelin protein was immunolocalised to the nucleus, the perinucleus and in cytoplasmic vesicles (Figure 10). GHS-R1 staining was observed in vesicle-like structures near the nucleus, in the perinucleus, throughout the cytoplasm and on the cell membrane (Figure 11). GOAT immunofluorescence staining was observed near the nucleus and in vesicle structures near the nucleus (Figure 12). Fluorescence staining of PC1/3 protein was observed near the nucleus and also in the in vesicles in the cytoplasm (Figure 13).

## Discussion

This is the first report demonstrating the expression of ghrelin, GHS-R1, GOAT and PC1/3 in human myometrium. There was an increase in prepro-ghrelin and GOAT expression at pregnancy, decreasing at labour onset, while GHS-R1 also decreased at labour. Furthermore, ghrelin processing was demonstrated at term pregnancy and in the non-pregnant state, in the myometrium. Other investigators have determined that total and/or acylated ghrelin levels in blood peak at mid-gestation and fall to their lowest levels at the third trimester [15-18]. Ghrelin receptor GHS-R1a mRNA expression in omental fat of pregnant women at term was found to be half that of lean and obese non-pregnant women [30]. Reduced ghrelin levels in third trimester maternal plasma have been hypothesised to be a response to marked changes in maternal energy intake [31]. However we did not determine ghrelin acylation status in the myometrium or circulating ghrelin levels. We did investigate ghrelin, GHS-R1, GOAT and PC1/3 expression in a small number of women with high BMI values however, there did not seem to be any correlation between high BMI levels and the expression of any of these proteins in the human myometrium at term labour or term non-labour. Increased circulating ghrelin levels in foetuses with intrauterine growth restriction (IUGR) [32], increased maternal plasma ghrelin levels observed in pregnancy-induced hypertension (PIH) leading to IUGR [15] and higher umbilical cord ghrelin plasma concentrations in small-for-gestational-age (SGA) neonates [33] all suggest ghrelin acts as a "hunger signal", with a role in foetal and neonatal energy balance. Decreasing circulating ghrelin levels contribute to physiological adaptation to the positive energy balance of pregnancy and reflect the nutrition status of pregnancy while decreased expression of locally synthesised and processed ghrelin may be involved in the preparation of the myometrium for labour.

There are two GHS-R isoforms: GHS-R1a has 366-amino acids with seven transmembrane regions, and GHS-R1b, which consists of 289-amino acids and is not activated by ghrelin. In humans GHS-R1a is present in the pituitary, thyroid, pancreas, spleen, myocardium and adrenal gland while GHS-R1b expression is even more widely expressed including amongst others, the skin, uterine fundus, ovary, testes and placenta [3,34]. Expression of mRNA for both GHS-R1a and b was identified in the myometrium, which had not been previously reported. GHS-R1 protein was localised to the cell surface and internally in vesicles. Other investigators previously established that GHS-R1 is recycled to the cell surface in HEK-293 cells after ghrelin binding and internalisation [35,36]. We have established that GHS-R1 protein expression increased in β-Estradiol-treated estrogen receptor-positive myometrial smooth muscle cells. Ghrelin expression was also regulated by estrogen in rat stomach [37], while β-Estradiol significantly stimulated the transfected human GHS-R promoter in rat pituitary cells with estrogen-responsive elements identified in the promoter region [38]. Estradiol-benzoate treatment of normal female or male rats up-regulated the expression of hypothalamic GHS-R1 [39]. Therefore, it is postulated that β-Estradiol increases the sensitivity of the myometrium to ghrelin, through increased GHS-R1 expression, thus contributing to the maintenance of uterine relaxation during pregnancy.

Murine GOAT has been described in the stomach, small intestine and colon tissues while human GOAT mRNA is expressed in the stomach, intestine, pancreas and chrondrocytes [8,9,40]. This is the first report of GOAT expression in the uterus, where it appeared to localise in the myometrial smooth muscle cells. There was increased myometrial GOAT expression observed at term pregnancy and a similar pattern of GOAT protein expression to pro-ghrelin mRNA and protein at term compared to labour was observed, suggesting GOAT activation and possible ghrelin acylation during pregnancy. Interestingly, other investigators have found that murine stomach GOAT mRNA correlated with circulating acylated ghrelin levels [41]. Yang *et al*. established that GOAT activity was inhibited by octanoylated ghrelin pentapeptides in GOAT-transfected insect cells, suggesting negative feedback regulation of acyl-ghrelin production [42], while in pituitary cells acylated ghrelin regulated its own production by increasing expression of GOAT mRNA [41]. However, more investigation is required to ascertain the mechanisms of GOAT regulation in the myometrium and consequently the impact on myometrial ghrelin processing. From these results we can deduce that it is possible that myometrial ghrelin may be octanoylated by GOAT in this tissue.

In addition to endocrine and neuroendocrine cells, PC1/3 expression has been reported in rat spleen macrophages and lymphocytes [43] however, this is the first report of PC1/3 expression in the uterus, and also its subsequent activation. This endoprotease is responsible for the conversion of pro-ghrelin to ghrelin *in vitro *and *in vivo *[11] and it has also been demonstrated that production of n-octanoyl modified ghrelin in cultured cells requires PC1/3, GOAT and n-octanoic acid [44]. Other investigators have demonstrated ghrelin and PC1/3 protein co-localisation in the mouse stomach [11]. There was increased PC1/3 protein expression in the myometrial samples acquired at hysterectomy due to premenopausal menorrhagia, compared to the pregnant myometrial samples. Menorrhagia results in the disruption of normal hormonal uterine regulation and thus increased PC1/3 protein may be responsible for the irregular pro-hormone processing evident in this condition. Additional examination of the involvement of PC1/3 in non-pregnancy related uterine abnormalities is therefore warranted. The presence of active PC1/3 and mature ghrelin strongly indicates a role for PC1/3 in the proteolytic cleavage of pro-ghrelin in the myometrium. However, further investigation is necessary into this and its' own activation in the myometrium. Subsequent analysis may also reveal information relating to the processing of other pro-hormones by PC1/3 in the uterus.

Parturition at term is an inflammatory process and intra-amniotic infection/inflammation is causally linked to preterm parturition [45]. Systemic administration of bacterial products to pregnant animals results in preterm delivery [46]. We have demonstrated that LPS administration decreased myometrial smooth muscle cell ghrelin protein. Interestingly, the bacterial polysaccharide LPS decreased plasma ghrelin protein levels from 3 to 12 hrs after administration in rats [47,48], and in healthy men induced an initial increase but rapidly decreased 5 hours after treatment [49]. Ghrelin itself exerts potent inhibitory effects on proinflammatory mediators via its actions on T cells, monocytes and endothelial cells [50-52]. In human endothelial cells it inhibits NF-κB activation *in vitro *and endotoxin-induced cytokine production *in vivo *[52]. Ghrelin inhibits the expression of Il-1β, IL-6 and TNF-α via GHS-R in human T cells while it's knockdown activates IκB in these cells, suggesting ghrelin plays a role in regulation of NF-κB activation [53,54]. This is significant given that NF-κB is a key transcription factor important in the regulation of inflammation, which plays a central role at labour [55]. The 5' upstream region from the start codon of the ghrelin gene contains 2 binding sites for NF-κB suggesting a possible feedback loop. Interestingly, the PC1/3 gene also contains two 2 putative NF-κB binding sites. As ghrelin has anti-inflammatory properties, decreasing ghrelin levels would enhance the overall inflammatory pathway occurring at labour. However, further work is necessary to determine if ghrelin is involved in NF-κB modulation and *vice versa*, in human myometrium.

## Conclusions

All the necessary machinery to produce active ghrelin is present in the myometrium, suggesting ghrelin may have an autocrine or paracrine effect in this tissue. Differential ghrelin gene expression at term pregnancy, the presence of ghrelin-processing enzymes and the *in vitro *contractility data indicates that it may play a role in the maintenance of uterine relaxation during pregnancy, and that at labour this inhibitory effect on myometrial contractility is down-regulated. Reduced local myometrial ghrelin production therefore may be involved in the preparation of the uterus for labour.

Furthermore, the regulation of ghrelin expression by significant modulators of myometrial contractility, β-Estradiol and bacterial lipopolysaccharide, represents a novel concept in terms of control of myometrial quiescience or activity during pregnancy, and at the time of labour. Finally, it is possible that pharmacological modulation of the ghrelin pathway in uterine smooth muscle may have future therapeutic potential.

## Competing interests

The authors declare that they have no competing interests.

## Authors' contributions

MOB conceived of the study, performed the tissue RNA and protein isolation, real time fluorescence tissue RT-PCR, tissue westerns, drafted the manuscript and performed the statistical analysis. PE did the cell culture treatments, protein isolation and cell westerns. JJM read and edited the final document. TJS acquired the funding, read and edited the final document. All authors read and approved the final manuscript.

## Authors' information

MOB: PhD. PE: MSc. Professor JJM: MB BCh, BSc. DCH, FRCOG, FRCPI consultant obstetrician and gynaecologist, chairman and head of the Department of Obstetrics and Gynaecology in UCHG, Galway. Professor TJS: PhD, vice-president for Research in NUI Galway.
